# Use of FGF-2 and FGF-18 to direct bone marrow stromal stem cells to chondrogenic and osteogenic lineages

**DOI:** 10.4155/fsoa-2016-0034

**Published:** 2016-09-22

**Authors:** Cindy Shu, Susan M Smith, Christopher B Little, James Melrose

**Affiliations:** 1 Raymond Purves Bone & Joint Research Laboratory, Kolling Institute, Northern Sydney Local Health District, St. Leonards, NSW 2065, Australia; 2 Sydney Medical School, Northern, The University of Sydney, Royal North Shore Hospital; 3 School of Biomedical Engineering, University of New South Wales, Kensington, NSW 2052, Australia

**Keywords:** adult mesenchymal stem cells, cartilage repair, chondrogenesis, intervertebral disc repair, low back pain, osteogenesis, spinal repair

## Abstract

**Aim:**

Intervertebral disc degeneration/low back pain is the number one global musculoskeletal condition in terms of disability and socioeconomic impact.

**Materials & methods:**

Multipotent mesenchymal stem cells (MSCs) were cultured in micromass pellets ± FGF-2 or -18 up to 41 days, matrix components were immunolocalized and gene expression monitored by quantitative-reverse transcription PCR.

**Results:**

Chondrogenesis occurred earlier in FGF-18 than FGF-2 cultures. Lower *COL2A1, COL10A1* and *ACAN* expression by day 41 indicated a downregulation in chondrocyte hypertrophy. *MEF2c, ALPL*, were upregulated; calcium, decorin and biglycan, and 4C3 and 7D4 chondroitin sulphate sulfation motifs were evident in FGF-18 but not FGF-2 pellets.

**Conclusion:**

FGF-2 and -18 preconditioned MSCs produced cell lineages which promoted chondrogenesis and osteogenesis and may be useful in the production of MSC lineages suitable for repair of cartilaginous tissue defects.

## Lay abstract

Intervertebral disc degeneration and low back pain is the number one global musculoskeletal disorder effecting 80% of the general population. A remedy for this condition is being eagerly sought as part of a WHO research priority. Stem cells are one potential therapy that shows promise in animal models, laboratory studies, and preclinical and early clinical trials. Conditioning of stem cells in the laboratory before injection may improve their efficacy for the alleviation of low back pain. In the present study we have developed a means of improving how stem cells form cartilage and bone, which should be of application in the repair of spinal defects.

**Figure 1.**
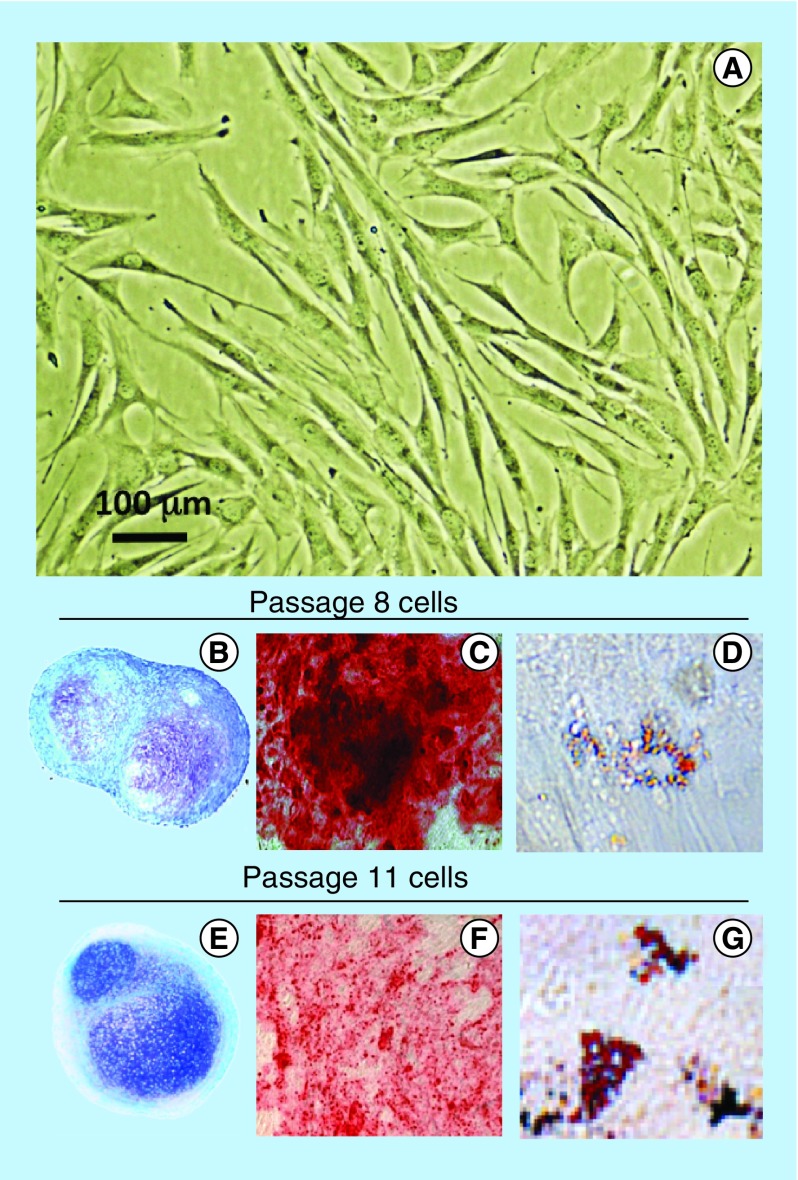
**Demonstration of the pluripotency of the ovine stromal mesenchymal stem cell preparation used in this study.** Morphological appearance of the mesenchymal stem cell preparation in monolayer culture **(A)** and demonstration of mesenchymal stem cell multipotency over passage 8 **(B–D)** and passage 11 **(E–G)**. Toluidine blue-stained cell pellets **(B & E)**, induction of an osteogenic phenotype using OsteoDiff selection media, calcium deposition evident by Alizarin Red staining **(C & F)**. Induction of an adipogenic phenotype in monolayer cultures in adipogenic selection media with characteristic fat droplet accumulation revealed by oil red-O staining **(D & G)**.

**Figure 2.**
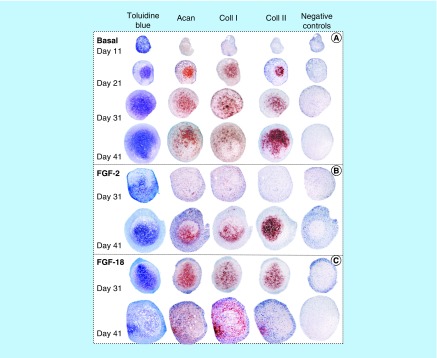
Histological localization of sulphated proteoglycan by toluidine blue staining and immunolocalization of aggrecan, type I collagen and type II collagen in sections of pellets cultured under basal culture conditions (ChondroDiff media) **(A)** and media supplemented with FGF-2 **(B)** or FGF-18 **(C)**. Acan: Aggrecan; Coll 1: Type 1 collagen; Coll 2: Type 2 collagen.

**Figure 3.**
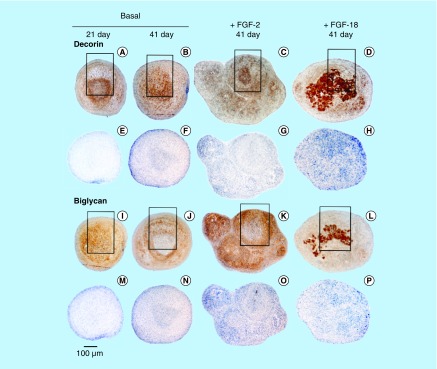
Immunolocalization of decorin **(A–H)** and biglycan **(I–P)** in micromass pellet cultures in basal medium (days 21 and 41) and in media supplemented with FGF-2 or -18. Rows **(E–H)** and **(M–P)** represent negative control sections for the specimens above them. The boxed areas indicated are shown at higher magnification in Figure 4.

**Figure 4.**
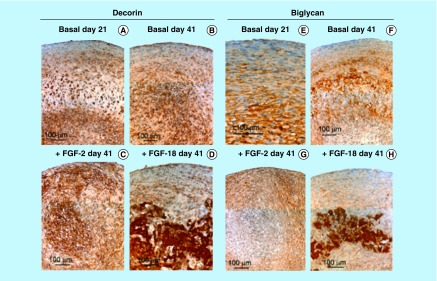
**Higher power images of decorin and biglycan immunolocalizations of the boxed areas of interest in Figure 3.**

**Figure 5.**
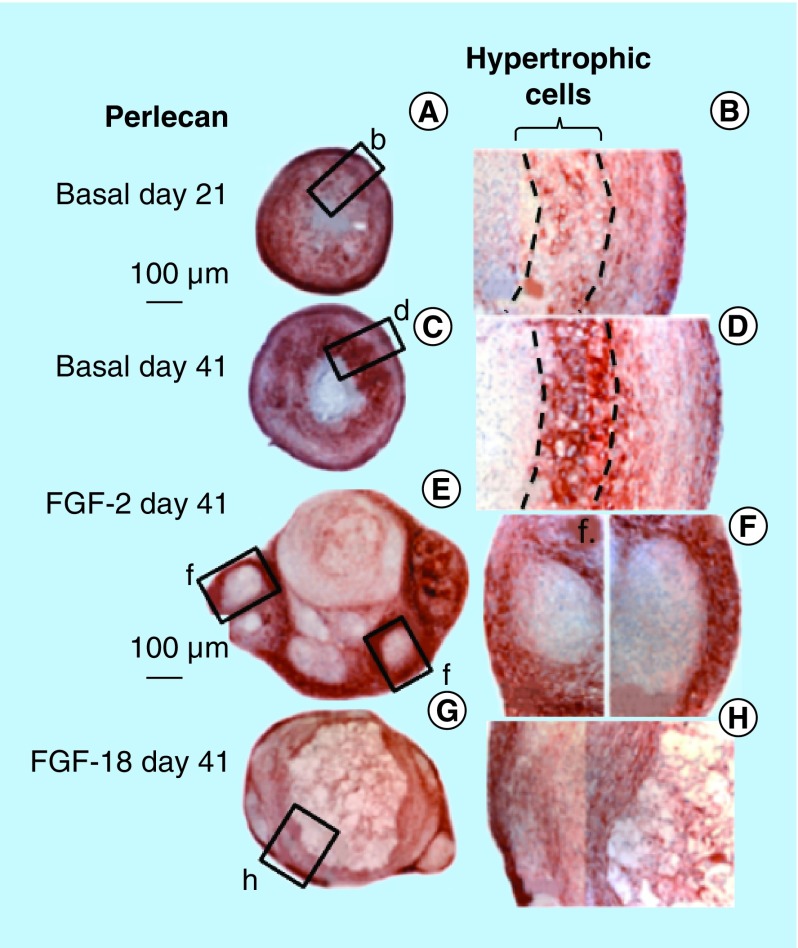
**Immunolocalization of perlecan in micromass cell pellets of the ovine bone marrow mesenchymal stem cell from basal days 21 (A) and 41 (C) cultures and those supplemented with FGF-2 or -18 (day 41 samples only [B & D]).** An area of hypertrophic cells deep in the cell pellet is indicated in **(B)** and **(D)**. FGF-2 **(E)** and -18 pellets **(G)** were significantly larger than the cell pellets cultured in basal media. The boxed areas in **(E & G)** are presented at higher magnification in **(F & H)**.

**Figure 6.**
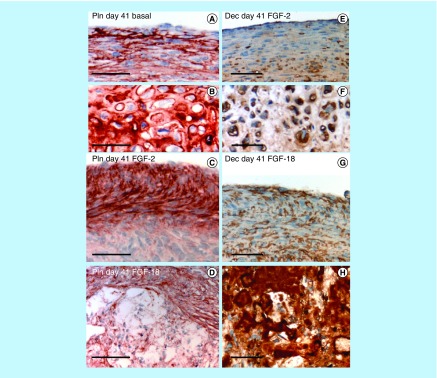
**Higher power images of selected areas in the pellets presented in Figures 3–5 demonstrating differences in the cellular organization in the pellets cultured under basal conditions or with FGF-2 or -18.** The cells in the pellet periphery are more flattened and smaller than cells deeper in the cell pellet, perlecan is prominently localized pericellularly in both cell type **(A & B)**. In FGF-2 cultures the outermost cells of the pellets **(C)** express more perlecan than the inner cells **(D)**. Decorin is also immunolocalized pericellularly around the peripheral **(E)** and deep cells **(F)** of the FGF-2 pellets; however, in the FGF-18 cultured cell pellets decorin is expressed by peripheral cells in the pellet **(G)** as well as being highly expressed around irregularly shaped extracellular matrix components **(H)**. Scale bars 100 μm. The red chromogen used is Nova Red, the brown chromogen is diaminobenzidine, cell nuclei counterstained with hematoxylin (blue). Dec: Decorin; Pln: Perlecan.

**Figure 7.**
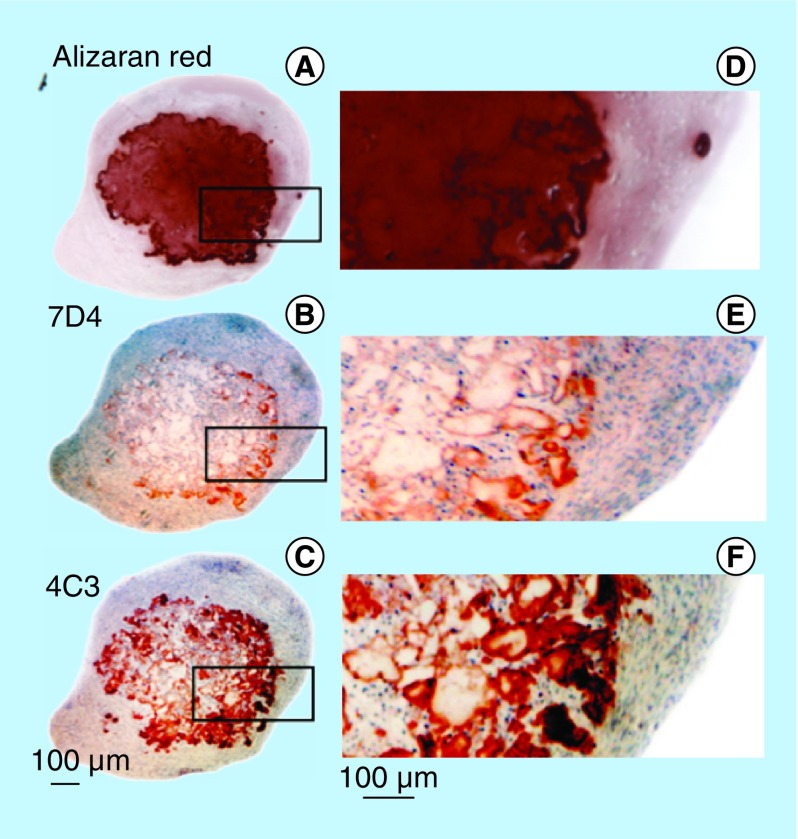
Demonstration of calcium deposition centrally in an FGF-18 cell pellet using Alizaran Red staining of the pellet section **(A)**. The CS sulphation motifs 7D4 **(B)** and 4C3 **(C)** are also depicted localizing to a similar area as the calcium deposition. Segments are serial sections through the same cell pellet. Higher power images of the boxed areas in **(A–C)** are provided in segments **(D–F)**.

**Figure 8.**
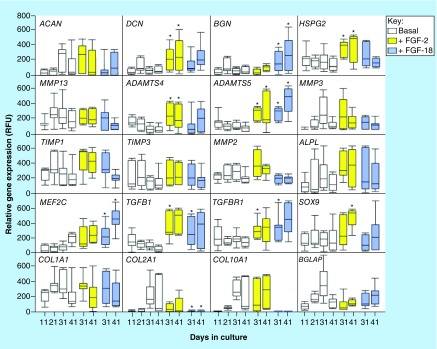
**Quantitative-reverse transcription-PCR of anabolic and catabolic matrix genes, and transcription factors using total RNA isolated from cell pellets of the basal, FGF-2 and -18 cultured ovine mesenchymal stem cells.** Box plots are presented. Mean values of six replicates are indicated by a solid bar, the upper and lower ranges of the values are also shown.

**Figure 9.**
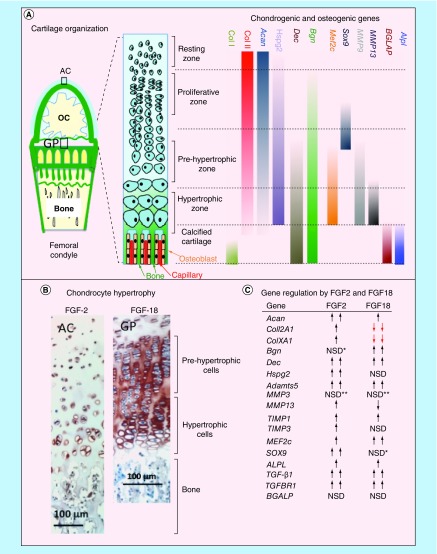
**(A)** Schematic depiction of the progressive changes in chondrocyte morphology through differentiation in the growth plate, and the chondrogenic and osteogenic genes, and the differentiation stages they affect. **(B)** Immunolocalization of FGF-2 pericellularly in articular chondrocytes and FGF-18 in the femoral epiphyseal growth plate clearly delineate the hypertrophic cells. **(C)** Summary of the anabolic and catabolic genes and transcription factors regulated by FGF-2 and -18 in pellet cultures of mesenchymal stem cells. NSD change not statistically different from basal conditions although and upward or downward trend may be evident as indicated. *Upward trend but not statistically significant. **Downward trend but not significantly significant AC: Articular cartilage; GP: Growth plate; NSD: Not significantly different; OC: Ossification center.

**Figure 10.**
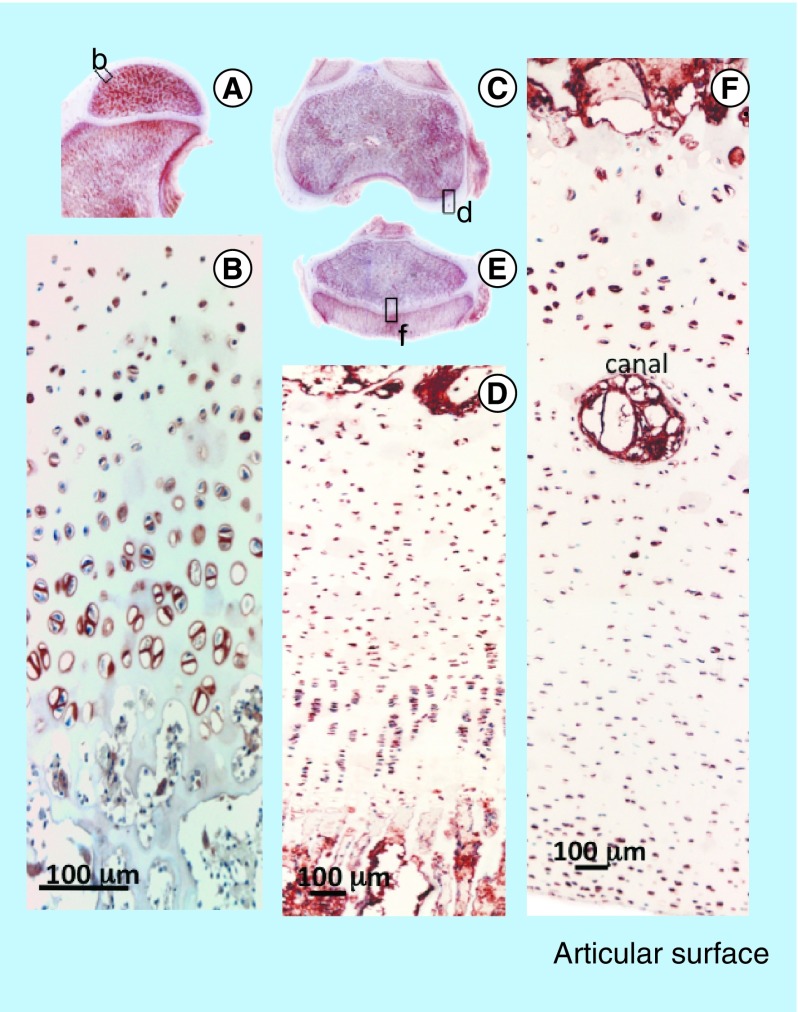
Immunolocalization of FGF-2 expressed by articular and growth plate chondrocytes in a newborn ovine hip **(A)** and femoral condyle **(C)** and tibial plateau **(E)** of the knee. FGF-2 is a pericellular component of articular chondrocytes **(B, D)** and epiphyseal growth plate chondrocytes **(F)**. FGF-2 is also strongly expressed in bone marrow **(A, C & E)**.

**Figure 11.**
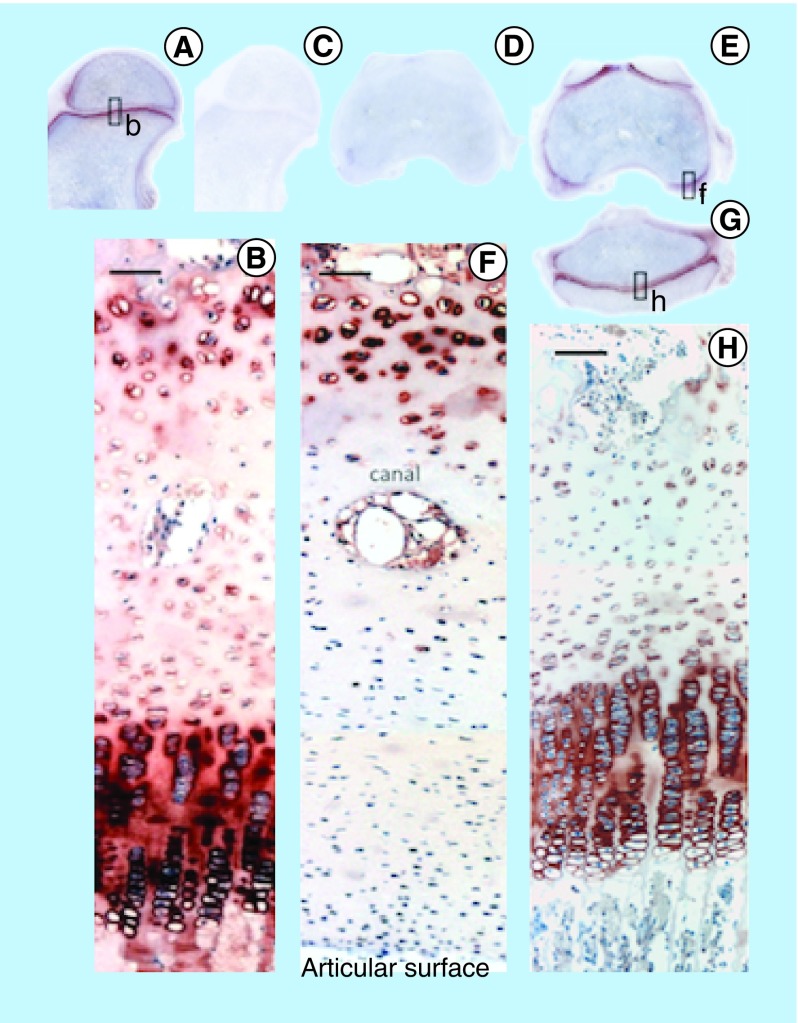
Immunolocalization of FGF-18 expressed by articular and growth plate chondrocytes in the hip **(A)** femoral condyle **(E)** and tibial plateau of the knee **(G)**. Perlecan is particularly prominently expressed by the deep hypertrophic chondrocytes in articular cartilage **(F)** and the columnar hypertrophic chondrocytes of the growth plates of the femur and tibia **(H)**.

The capacity of mesenchymal stem cells (MSCs) for self-renewal and directed differentiation [1,2] makes them attractive candidates for a range of cell-based therapies [3–5], and considerable interest has centered around their use in regenerative medicine and in intervertebral disc (IVD) repair [6–8]. MSCs have been sourced from bone marrow [9,10], adipose tissue [11], synovium [12], olfactory [13] and fetal spinal tissues [14]. In the present study we utilized MSCs isolated from adult ovine bone marrow and stimulated these with FGF-2 and -18 to examine if we could direct the chondrogenic and osteogenic differentiation of the MSCs *in vitro*. FGF-2 upregulates *Sox9* during cellular expansion of chondroblasts and early activation of chondrogenesis, and augments extracellular matrix (ECM) synthesis [15]. FGF-18 signaling through FGFR3 modulates the expression profiles of established chondrocytes during chondrogenesis, delaying hypertrophy but enhancing anabolic ECM gene expression during early chondrogenesis [16,17].

The FGF family currently has 23 members, which signal through four FGFRs that each occur as three alternatively spliced isoforms and are important in skeletogenesis [18]. FGFR1c, FGFR2c and FGFR3c are the isoforms used by mesenchymal cells in cartilage and bone development whereas epithelial cells in the ectoderm signal through FGFR2b. In early limb bud development prior to the establishment of the cartilaginous rudiment, FGFR1c is widely expressed, however, as the limb bud develops FGFR2c is expressed in the central mesenchymal condensation. Chondrocyte differentiation ensues as the limb bud develops into the cartilaginous rudiment along with expression of FGFR3c [19]. During joint development, chondroprogenitor cells express FGF-1, -2, -9 and -18 and utilize FGFR1c, FGFR2c, FGFR3c and perlecan to undertake cell signaling and initiate cell proliferation and ECM production, which enlarges the cartilaginous rudiment *in vivo* [18]. Studies with Baf-32 engineered cells expressing individual FGFR isoforms have demonstrated FGF-2 and -18 utilize FGFR1c and FGFR3c to promote chondrogenesis *in vitro* [20,21]. Widespread FGF-2 expression occurs in human fetal spinal development at 12–20 weeks gestation, FGF-2 primes the discoprogenitor cells for chondrogenesis, which is a major driving force in early spinal development [22–24]. FGF-18 signals through FGFR3c within the cartilaginous vertebral rudiment promoting hypertrophy and establishment of the primary ossification center in the vertebral rudiments. These subsequently undergo ossification by a process similar to endochondral ossification to form the spinal vertebrae [23].

Cartilaginous tissues such as articular cartilage (AC), knee-joint meniscus, IVD and tendon, all contain collagens and proteoglycans to variable degree as functional ECM components equipping these tissues with the ability to withstand tensional stresses or to act as weight-bearing structures located at strategic points in the axial skeleton. With aging, trauma and disease these ECM components are degraded by a family of 26 MMPs, ADAMTS and ADAMS metalloprotease families [25]. This can compromise the normal biomechanical properties of these tissues and lead to secondary changes in adjacent tissues in joint structures resulting in impaired articulation, diminished mobility, pain generation and a reduction in the quality of life. The IVD is a particularly important supportive structure in the axial skeleton. It is a composite tissue consisting of an outer annulus fibrosus containing annular lamellae rich in type I collagen, which provides tensile strength [24,26]. The annulus fibrosus also contains type II collagen in its inner regions. The central region of the IVD is the nucleus pulposus (NP). This region is rich in aggrecan, which forms massive ternary complexes with hyaluronan (HA) and link protein which are entrapped in the NP within a network of type II collagen fibers. The aggrecan–HA macroaggregates have impressive water regain properties equipping the NP and composite IVD with hydrodynamic weight-bearing properties. Thus the IVD has both the ability to withstand tension and compressive loading of the spine and its properties are coordinated with other components in the axial skeleton critical to the provision of a supportive framework. Articular cartilage (AC) is another important supportive weight-bearing tissue in human joints and also provides joint articulation. Type II collagen and aggrecan are significant functional ECM components in AC [27]. The semi-lunar fibrocartilaginous menisci of the knee joint contains type I collagen and lesser amounts of type II collagen and aggrecan, and has important weight-bearing properties and also protects the weight-bearing regions of the femoral and tibial AC from damaging focal overload [28]. The tendons and ligaments in the human body interconnect bone to muscle and bone to bone and are rich in type I collagen, which provides tensional strength. Ligaments and tendons have important roles in the stabilization of joint structures and in the transmittance of forces from the muscles that facilitate joint movement. As already mentioned, all of the aforementioned tissues can deteriorate due to excessive proteolytic attack in disease processes and with traumatic loading. Cells of a chondrogenic background are responsible for the assembly of these tissues and are also important in tissue homeostasis in maturity, however, they have a limited ability to affect tissue-repair processes. Adult stem cells represent a means of overcoming this shortcoming and show considerable therapeutic promise in these tissues. It was from this background that we embarked on the present study using FGF-2 and -18 to develop adult stem cell lineages with chondrogenic and osteogenic capability. This work follows on from earlier basic studies on human fetal spinal development where FGF-2, -18, the heparan sulphate proteoglycan perlecan and FGFR1c and FGFR3c were all prominently expressed by the chondroprogenitor cell populations [23]. Chondrogenesis is an active driving force promoting early spinal development. It was, therefore, logical to follow-up on these findings by determining if adult stem cells could be primed to develop into chondrogenic and osteogenic cell lineages, which may be applicable to the repair of cartilaginous tissues including the IVD. Besides its ability to act as a low-affinity co-receptor for FGFs, perlecan is also an early chondrogenic marker with an extensive repertoire of interactive matrix components through which it provides ECM stabilization. Thus the expression of perlecan by the adult stem cells was of interest in our strategic plans aimed at disc repair. The IVD is a particularly important tissue to look at in such repair strategies. Degeneration of the IVD is associated with low back pain (LBP), which is now considered to be the number one musculoskeletal global condition in terms of years lived with disability, loss of working days, impairment in the quality of life and detrimental socioeconomic impact of global significance. The American Academy of Pain Medicine stated in 2016 that chronic pain costs in the USA were $560–635 billion/annually and that 53% of all chronic pain patients had LBP with 31 million people suffering from this condition at any one time [7]. Crow and Willis (2009) quoted US$317 billion costs for LBP for the USA in 2009 [8]. The WHO indicated that the development of MSCs and bioscaffolds to promote IVD repair should be made high-priority research objectives and many scaffold and cell-based treatment strategies have subsequently been developed for the treatment of LBP [29]. The present study has developed adult stem cell lineages which deserve further investigation in such strategies for their ability to promote disc repair, alleviation of LBP and the repair of cartilaginous tissues in general.

## Materials & methods

ChondroDiff^®^ (cat no. 130-091-679), OsteoDiff^®^ (cat no. 130-091-678) selection medias were purchased from Miltenyi Biotec (North Ryde, New South Wales, Australia). Chondroitinase-ABC, dexamethasone, indomethacin and 3-isobutyl-1-methylxanthine were obtained from Sigma-Aldrich (New South Wales, Australia). Menzel and Glaser SuperFrost ultraPlus, positively charged microscope slides were obtained from Fisher Scientific, GmbH, Braunschweig, Germany. NovaRED substrate was obtained from Vector Laboratories (CA, USA). FGF-2 (cat no. 100-18C) and FGF-18 (cat no. 100-28) were purchased from PeproTech (Sapphire Biosciences, New South Wales, Australia).

### Molecular biology

cDNA primers were manufactured by Sigma Genosys (New South Wales, Australia), PCR reagents were purchased from Invitrogen (Victoria, Australia). RNA isolation kits and Omniscript for PCR were purchased from Qiagen (Victoria, Australia). RNase inhibitor and Immomix were purchased from Bioline (Sydney, Australia). SYBR Safe^®^ was purchase from Invitrogen. Genomic DNA isolation kits were purchased from Qiagen. A disposable pellet pestle (Kimble Kontes) and cordless motor for RNA isolation were purchased from Sigma Aldrich.

### Antibodies

Anti-type I collagen (clone I-8H5, cat no. 0863170) and anti-type II collagen (clone II-4CII, cat no. 08631711) were purchased from MP Biomedicals (OH, USA). Rat monoclonal antibody A7L6 to perlecan domain IV (cat no. ab2501) was obtained from abcam through Sapphire Biosciences (Redfern, Australia). A rabbit polyclonal antibody (pAb) # 2194 to aggrecan G1 domain was a gift from Dr J Mort, Joint Diseases laboratory, Shriners, Hospital for Children, McGill University, Montreal, Quebec, Canada [30]. pAb 2194 was raised against a mixture of four aggrecan-specific G1 peptide–ovalbumin conjugates including HDNSLSVSIPQPSGGC, RVLLGTSLTIPCYFIDPMHPVTTAPS, TEGRVRVNSAYQDKGGC and SSRYDAICYTG (single-letter amino acid code). Rabbit pAb to C-terminal peptide sequences of decorin ([CGG]YVRSAIQLGNYK) and biglycan ([CGG]TDRLAIQFGNYKK) were also prepared by EZ Biolab (IN, USA). The GGC tripeptide was added as a linker (GG) and the cysteine (C) for coupling of ovalbumin to increase antibody titre. These pAbs were affinity purified by solid-phase absorption on peptide affinity columns prepared from the immunizing peptides. Conditioned media was collected from 4C3 and 7D4 hybridoma cultures as a source of these monoclonal antibodies, aliquoted and stored frozen at -20°C till required. Rabbit antihuman FGF-18 (cat no. HPA018795) was obtained from Sigma-Aldrich (Sydney, Australia).

### Tissues

Ovine knee and hip tissues were harvested from newborn lambs supplied by a local abattoir. Adult (18 month) male wethers were used for bone marrow harvest.

### Bone marrow harvest

Ovine iliac crest bone marrow aspirates of three pooled ovine male wether donors were collected under general anesthesia into Na_2_EDTA. All animal procedures were approved by our Institutional Animal Care and Ethics Committee.

## Methods

### Isolation, expansion & authentication of ovine bone marrow MSCs

The MSCs isolated complied with the minimal criteria for defining multipotent MSCs as described by The International Society for Cellular Therapy [30]. That is, they adhered to tissue culture plastic; they expressed high levels of CD44 and CD106, but were negative for CD11b, CD34, CD45; and they were multipotent cell types with the capability under appropriate growth conditions of differentiating into osteoblasts, adipocytes and chondroblasts and displayed multipotency up to passage 11. The findings reported in the present study were from passage 8 cells. Ovine iliac crest bone marrow aspirates were collected into Na_2_EDTA and the buffy coat cells were collected by centrifugation at 2500 × *g* for 10 min [31] and resuspended in phosphate buffered saline (PBS; 1.5 ml), then filtered through a cell sieve (70 μm) to remove cellular and tissue debris and the clarified cell suspension layered on to a Ficoll gradient (10 ml). The Ficoll gradient was centrifuged at 1200 × *g* for 20 min in a swing-out rotor and the cells at the interface were collected and washed twice in sterile PBS. The buffy coat cells were allowed to attach overnight to 75 cm^2^ canted neck polystyrene tissue culture flasks in Dulbecco's modified Eagle medium (DMEM) supplemented with 10% fetal bovine serum (FBS), antibiotics and 2 mM L-glutamine (DMEM-FBS culture medium). The flasks were subsequently washed with PBS to remove nonadherent cells and the adherent cells were further cultured in DMEM-FBS till confluent then serially passaged up to passage 11 to expand cell numbers. The expanded cells were examined by flow cytometry using the anti-ovine markers CD44, CD34, CD45, CD11b and antihuman CD106. The MSCs isolated were CD44 and CD106 positive, but CD34, CD45 and CD11b negative. The MSCs were frozen down in liquid nitrogen in aliquots of 5 million MSCs/cryovial in DMEM + 20% FBS + 10% v/v DMSO.

### Demonstration of MSC multipotency

The MSCs were grown in micromass pellet and monolayer culture in chondrogenic, adipogenic and osteogenic selection medias to demonstrate chondrogenesis, adipogenesis and osteogenesis.

### Monolayer culture of MSCs

The MSC numbers were initially expanded in 75 cm^2^ canted neck flasks in DMEM + 10% FBS till 75–80% confluent then the cells were harvested by trypsinization and cell numbers determined on a hemocytometer using trypan blue exclusion.

### Monolayer adipogenesis cultures

The MSCs were seeded into 12-well plates (13,150 cells/cm^2^) and cultured in DMEM + 10% FBS with a media change once per week. When the cells had reached 70–80% confluence, the media was changed to DMEM supplemented with 10% FCS, dexamethasone (0.5 μM), indomethacin (50 μM) and 3-isobutyl-1-methylxanthine (0.5 μM), and culturing continued for a further 7 days. The monolayers were fixed in 10% neutral buffered formalin for 30 min and washed in PBS. The fixed cells were stained with Oil red-O stain (5 μg/ml) in 70% isopropanol for 1 h at 37°C.

### Monolayer MSC osteogenesis cultures

The MSCs were cultured in 24-well plates at a density of 13,150 cells/cm^2^ and cultured in NH OsteoDiff selection media with media changes every 3 days. The cultures were terminated on day 18 and stained with Alizarin Red S to demonstrate calcium deposition. The cells were initially washed in calcium-free cold PBS then fixed in 10% neutral buffered formalin for 15 min, rinsed in PBS then distilled water and stained with a 1% w/v solution of Alizarin Red S pH 4.2 for 15 min followed by rinsing in several changes of distilled water [32].

### Micromass chondrogenesis pellet cultures of MSCs

Pellet cultures were established by dispersing 250,000 MSCs in 0.4 ml of ChondroDiff selection media in a 15 ml conical test tube. The cells were spun down and cultured in ChondroDiff selection media at 37°C under an atmosphere of 5% CO_2_ in air with 98% humidity, media was changed every 3 days. After 21 days, the cultures were either continued in ChondroDiff selection media or in ChondroDiff media supplemented with FGF-2 or -18 (20 ng/ml) for an additional 20 days and the cultures were terminated on day 41. Chondrocyte pellets were removed after various culture periods, washed in PBS and fixed in 10% neutral-buffered formalin then processed to paraffin and microtome sections attached to positively charged microscope slides. These were stained with toluidine blue-fast green to visualize tissue proteoglycans. Type I and II collagen and aggrecan were also immunolocalized to confirm the chondrogenic status of the cells [33]. Staining patterns in the monolayer and pellet cultures were documented on a Leica DFC 450 digital photo microscope system (Leica Microsystems Pty Ltd, North Ryde, Australia).

### Quantitative-reverse transcription PCR gene profiling of MSC pellets

Chondrocyte pellets (n = 5–7) from micromass MSC cultures in ChondroDiff media; or in ChondroDiff media supplemented with FGF-2; or FGF-18 were collected on days 31 and 41. On day 21 the media was supplemented with FGF-2 or -18 and culturing continued. Pellets from the FGF cultures were harvested on days 31 and 41. The pellets were homogenized using pellet pestles driven by a cordless motor. Total RNA was extracted using Trizol (Invitrogen) and purified using a Qiagen RNA extraction kit and quantitated as previously described [34]. RNA (1 μg) from each sample was reverse transcribed (Omniscript; Qiagen) using random pentadecamers (50 ng/ml, Sigma-Genosys) and RNase inhibitor (10 U per reaction, Bioline, Sydney, Australia). The resulting cDNA was subjected to real-time PCR in a Rotorgene 6000 (Corbett Life Science, New South Wales, Australia) using Immomix (2× dilution; Bioline), SYBR Green I (10,000×- dilution; Cambrex Bioscience) and 0.3 μM validated ovine-specific primers (Table 1). Standard curves were constructed using a range of dilutions of total ovine MSC cDNA, and a relative copy number determined for each gene of interest. Melt curves were obtained following quantitative-reverse transcription (qRT)-PCR to check for single products, specificity was confirmed by sequencing at Sydney University Prince Alfred Macromolecular Analysis Centre. The genes investigated, primers, annealing temperatures used for qRT-PCR are listed in Table 1. The qRT-PCR data are presented as box plots showing interquartile (25–75%) range with whiskers indicating the maximum–minimum range and the mean values indicated by a horizontal line within the box.

### Histochemical processing of cell pellets

Pellets from six cultures for each growth condition were fixed in 10% neutral buffered formalin for 12 h, dehydrated through sequential alcohols, cleared in chloroform then infiltrated and embedded in paraffin by our standard procedures [23,26,35]. The pellets were sectioned at 4 μm and immunolocalizations undertaken for type I and II collagen, perlecan, decorin, biglycan and the 4C3 and 7D4 CS sulphation motifs. Pellet sections were also stained with toluidine blue-fast green to visualize anionic proteoglycans and with Alizarin Red S to visualize calcium deposition.

### Histological processing of ovine knee & hip tissues

Ovine newborn knee and hip joints were fixed en bloc in 10% neutral buffered formalin and decalcified in 10% formic acid/5% formalin then 4 μm sections prepared. The hip joints were sectioned in the midsaggital plane while a mid-coronal section through the main weight-bearing regions of the femoral condyle and tibial plateau were also prepared.

## Histochemistry

Anionic proteoglycans were localized in tissue sections by staining for 10 min with 0.04% w/v toluidine blue in 0.1 M sodium acetate buffer, pH 4.0 followed by a 2-min counterstain in 0.1% w/v fast green FCF.

### Immunohistochemistry

Incubations with primary antibodies were performed using a Sequenza vertical cover-plate immunostaining system (Thermo Scientific Shandon, Cheshire, UK). Endogenous peroxidase activity was blocked by incubating the tissue sections with 0.3% H_2_O_2_ for 5 min and after washing in water nonspecific binding sites were blocked with 10% swine serum for 10 min. Sections destined for localization of type I, II collagen were predigested with bovine testicular hyaluronidase (1000 U/ml) for 1 h at 37°C in phosphate buffer pH 5.0 prior to incubation with primary antibody. The primary, anti-type I and II collagen (1/200 dilution) antibodies were diluted in Dako (Dako, Sydney, Australia) diluent (cat no. S202230) and applied to the slides and incubated at 4°C overnight. The primary antibodies were subsequently localized using Dako Envision+, an horse radish peroxidase-labeled polymer and anti-mouse (cat no. K4001) and -rabbit (cat no. K4004) antibodies for the visualization of the tissue immune complexes using Nova RED substrate for color development. Negative control sections were also prepared where the authentic primary antibody was replaced with an irrelevant isotype-matched mouse IgG directed against *Aspergillus niger* glucose oxidase, an enzyme which is neither present nor inducible in mammalian tissues or to a concentration-matched rabbit nonimmune serum sample.

### Statistical analyses

RNA was isolated from a total of five to seven pellets for each gene. Non-Gaussian qRT-PCR data were analyzed by Mann–Whitney U ranked tests. The α-level was set at 0.05. Gene expression data were presented as box plots showing interquartile (25–75%) range, with whiskers indicating the maximum–minimum range and mean values were indicated with a horizontal line within the bar. Values that were significantly higher or lower than the basal culture data on day 21 were labeled with an asterisk (p < 0.05).

## Results

The MSCs isolated by the Ficoll procedure had typical fibroblastic morphologies in monolayer culture (Figure 1A). The multipotency of the MSC preparations was established in pellet cultures in ChondroDiff differentiation media (Figure 1B & E) and in monolayer cultures in OsteoDiff (Figure 1C & F) and Adipogenic selection medias (Figure 1D & G), and maintained up to passage 11. Passage 8 cells were used in this study.

Microscopic examination of the micromass pellet sizes under basal culture conditions (ChondroDiff media only) and in media supplemented with FGF-2 or -18 showed that the FGF-2 and -18 pellets were significantly larger from day 21 (Figure 2). Toluidine blue-stained proteoglycan, aggrecan and type I and II collagen immunolocalizations of sections of pellets demonstrated the chondrogenic phenotype inducible in the MSCs under these culture conditions (Figure 2B & C). FGF-2 and -18 both promoted chondrogenesis with FGF-18 eliciting an earlier response than FGF-2 on day 31 while FGF-2 exhibited a strong chondrogenic response on day 41. FGF-18, however, did not maintain type II collagen deposition but induced type I collagen production on days 31–41, consistent with the induction of an osteogenic phenotype (Figure 2C).

Decorin (Figure 3A–D) and biglycan (Figure 3I–L) were also immunolocalized in pellet cultures under basal conditions over days 21–41 and in FGF-2 and -18 supplemented cultures (Figure 3). Decorin and biglycan had a widespread immunolocalization pattern throughout the pellets in the basal and FGF-2 cultures, however, they were particularly strongly localized in a central region of the pellet in the FGF-18 cultures (Figure 4D & H). Perlecan had a differing immunolocalization pattern to that of decorin or biglycan in the pellets displaying a predominantly peripheral distribution under basal conditions over days 21–41 and in cultures supplemented with FGF-2 or -18 (Figure 5A, C, E & G). Some evidence of multiple clone formation was evident in the FGF-2 and -18 cultures (Figure 5E & G) with perlecan displaying a predominant distribution around the margins of each discrete population of cells (Figure 5B, D, F & H). Examination of selected areas of the pellets under higher power magnification demonstrated perlecan had a widespread pericellular distribution around the more flattened cells at the periphery of the pellet in the basal and FGF-2 cultures (Figure 6A & C), and had a prominent pericellular localization pattern around the deeper located hypertrophic cells (Figure 6B) in the basal cultures on days 31–41; however, in FGF-2 and -18 cultures perlecan immunolocalization was reduced in this central region (Figure 6C & D). Thus the distribution of perlecan in MSC pellet cultures differed quite markedly from that of decorin and biglycan. Examination of higher power immunolocalizations of decorin in FGF-2 and -18 pellets on day 41 demonstrated markedly different localization patterns (Figure 6E–H). Decorin had a pericellular distribution around the outer and inner hypertrophic cells in the FGF-2 pellets (Figure 6E & F); however, in the FGF-18 pellets decorin had a very strong localization in the central region of the pellet (Figure 6H). Calcium deposition visualized by Alizaran Red in the FGF-18 pellets was also prominent in this central region of the pellet (Figure 7A & D). The CS sulphation motifs 7-D-4 (Figure 7B & E) and 4-C-3 (Figure 7C & F) also displayed a strong localization pattern in this central area of mineral deposition.

Comparative gene profiling of cells from each of the culture conditions (Figure 8) showed that decorin and biglycan gene expression were elevated in the FGF-2 and -18 cultures, *ACAN* was maintained and type II collagen gene expression markedly decreased by 41 days of culture. Type X collagen expression was elevated in the basal and FGF-2 cultures on day 41 but undetectable in the FGF-18 cultures over days 31–41 indicating that FGF-18 downregulated chondrocyte hypertrophy. Perlecan expression was maintained in the FGF-2 cultures but downregulated by FGF-18. Expression of *SOX9* and TGFB1/TGFBRI were maintained or were slightly elevated in the FGF-2 and -18 cultures, consistent with induction of a chondrogenic phenotype. Myocyte-specific enhancer factor 2C also known as MADS box transcription enhancer factor 2, polypeptide C (*MEF2C*), a transcriptional regulator of chondrocyte hypertrophy and osteogenic differentiation was upregulated in the FGF-18 cultures on days 31 and 41; however, the downregulation of type X and type II collagen gene expression to virtually undetectable levels and a lowering of *MMP13* expression indicated that FGF-18 delayed hypertrophy. *ALPL* expression was maintained in the FGF-2 and -18 cultures while *BGLAP* expression was not significantly different from expression levels in the basal cultures and showed a steady increase over days 21–41. BGLAP or γ-carboxyglutamic acid-containing protein (BGLAP), also known as osteocalcin, is a highly abundant bone protein secreted solely by osteoblasts, encoded by the *BGLAP* gene. Its Gla domain interacts with calcium and hydroxyapatite promoting bone mineralization and calcium ion homeostasis, along with *ALPL, BGLAP* represents a useful early marker of early bone formation. *ADAMTS4* and *ADAMTS5* were both upregulated in the FGF-2 cultures and *ADAMTS5* in the FGF-18 cultures. *MMP-13*, a marker of chondrocyte hypertrophy was significantly downregulated by FGF-18 in agreement with type X collagen expression. Type X collagen and MMP13 are both markers of the hypertrophic chondrocytic phenotype.

A schematic of the morphology of the epiphyseal growth plate cartilage and its constituent chondrocytes and the marker genes operative at various stages of chondrocyte differentiation was prepared to summarize this process (Figure 9A). *COL1A1, BGLAP and ALPL* are all operative in the terminal stages of growth plate differentiation in the calcified cartilage while *MMP9, MMP13* and *MEF2c* are expressed in the prehypertrophic and hypertrophic chondrocytes. *DEC* and *BGN* are expressed throughout the chondrocyte maturation process from the proliferative phase through to the calcified cartilage. *SOX9* directs the early chondrogenic differentiation of the chondrocytes in the proliferative/prehypertrophic stages. *COL2A1, ACAN* and *HSPG2* are prominent chondrogenic marker genes acting throughout the resting to hypertrophic phases of chondrocyte differentiation. *TGFB1* is also an early chondrogenic regulatory gene. Immunolocalization of FGF-2 identified its pericellular immunolocalization in the articular chondrocytes and prominence in the deep hypertrophic cells (Figure 9B) while FGF-18 was prominently expressed by the columnar prehypertrophic and hypertrophic epiphyseal chondrocytes in the growth plate (Figure 9B). A table of the anabolic and catabolic genes, and transcription factors regulated in prechondrogenic and chondrogenic cells by FGF-2 and -18 was assembled to summarize the major findings of the present study (Figure 9C). Anabolic chondrogenic genes such as *ACAN, COL2A1, HSPG2* and *SOX9* were all upregulated by FGF-2 consistent with its role as a promoter of chondrogenesis. In contrast, while FGF-18 promoted early chondrogenic gene expression on day 31, by day 41, FGF-18 also significantly downregulated Coll2A1 and the hypertrophy marker genes *COL10A1*, *COL2A1* and *MMP13*, and upregulated genes which promote osteogenesis (*MEF2C*, *ALPL*, *TGFB1*). *DEC* and *BGN* were also upregulated by FGF-18 and their deposition in the cell pellets in areas of calcium deposition supported potential roles in biomineralization. Decorin and biglycan also control the bioavailability of TGF-β in tissues thus they may have a role in the regulation of chondrogenesis and promotion of osteogenesis.

Comparative immunolocalization of FGF-2 and -18 in newborn ovine stifle and hip joints (Figures 10 & 11) demonstrated different distribution patterns. FGF-2 was strongly localized in the marrow space and the pericellular matrix of articular chondrocytes in the hip and knee (Figure 10). In contrast, FGF-18 was predominantly expressed by the columnar pre- and hypertrophic chondrocytes of the growth plates with relatively weak expression by the articular chondrocytes and little or no expression in the marrow space (Figure 11). This was consistent with FGF-2 promoting and maintaining chondrogenesis and FGF-18 promoting early chondrogenesis, chondrocyte maturation and the promotion of osteogenesis in joint tissues.

## Discussion

### MSCs & tissue repair

Adult multipotent stem cells hold tremendous promise in regenerative medicine and developmental biology and specifically in joint repair [2,4–5,36–37]. A major challenge in their therapeutic application lies in how their differentiation is controlled to facilitate production of cell lineages of defined properties, engraftment at the therapeutic site of action and maintenance of their viability throughout their therapeutic period. The aim of the present study was to examine whether FGF-2 and -18 could be used to select chondrogenic and osteogenic cell lineages. These experiments extended our earlier observations on spatiotemporal changes in ECM composition and the immunolocalization of FGF-2 and -18 in human fetal spinal development [23]. In the present study, FGF-2 and -18 stimulated ECM production in the pellet cultures; the FGF-2 pellets were larger than the FGF-18 pellets, however, both were significantly larger than the pellets grown under basal conditions. FGF-2 has been reported to stimulate human bone marrow-derived MSC proliferation and delay the loss of chondrogenic potential through the transient expression of JNK P13K–Akt and ERK-1, -2 cell-signaling pathways [38,39]; FGF-2 also inhibits lineage differentiation of MSCs, by delaying Erk-1 and -2 phosphorylation and represses osteogenic gene expression during osteoinduction *in vitro* [38]. This may explain the absence of Alizarin Red staining in the FGF-2 stimulated cell pellets in the present study.

### Induction of TGF-β1/TGFBR1 by FGF-2 & -18 & their roles in chondrogenesis & bone formation

The micromass pellet culture system is commonly used to examine the chondrogenic differentiation of MSCs. Initial studies involved the use of TGF-β1 as an induction agent for *in vitro* chondrogenesis of MSCs [40], subsequent studies showed that TGF-β2 and -β3 were even more effective chondrogenic agents [41]. An analysis of molecular markers of *in vitro* chondrogenesis by bone marrow MSCs demonstrated distinct stages of chondrocyte differentiation toward full chondrocyte commitment (Chen *et al*.). The earliest stages of *in vitro* chondrogenesis were an upregulation of TGF-β1, -β2 and -β3 followed by upregulation of the BMP and SMAD pathways to reach the final stages of chondrocytic commitment [42,43]. This is followed by maturational changes in the chondrocytes leading to hypertrophy, apoptosis, cartilage calcification, penetration of blood vessels and bone formation. The initial stages of TGF-β-induced chondrogenesis involve the rapid deposition of cartilage-specific ECM components such as collagen II, aggrecan and perlecan.

TGF-β is synthesized in the endoplasmic reticulum attached to a prodomain latency-associated peptide, which prevents its interaction with TGF-β receptors. The latency-associated peptide domain of TGF-β interacts with the RGD-binding domains of latent TGF-β-binding proteins (LTBP-1, -3, -4) to form an inactive TGF-β complex which is deposited in the tissues [44] and is also interactive with the Arg–Gly–Asp-binding integrins, α β1, α β3, α β5, α β6, α β8 and α8β1. Fibrillin-1, -2, -3 also share structural homology with the latent transferring growth factor β-binding proteins (LTBPs) and are critical for the placement of latent TGF-β within tissues in close proximity to the cells that assemble these polymers. This is a critical step in tissue homeostasis, evidenced by mutations in fibrillin-1, which result in Marfan syndrome. Fibrillin/LTBP assemblies have roles in the delivery of TGF-β to cells in appropriate amounts in a spatiotemporal-directed manner, which is important not only in tissue development but also in the homeostasis of mature tissues facilitating cross-talk among TGF-β signaling pathways, integrins and the ECM [44].

In the present study, FGF-2 and -18 significantly upregulated *TGFB1* and *TGFBR1* gene expression on days 31–41. This was accompanied by the deposition of chondrocyte-specific ECM components, which were immunolocalized in the MSC cell pellets. FGF-18 elicited an earlier chondrogenic response than FGF-2, but by day 41 had downregulated *COL2A1* and *COL10A1* expression to virtually undetectable levels. FGF-18 also downregulated *MMP13* on day 41, thus after an initial chondrogenic phase, chondrocyte hypertrophy was also downregulated. Preconditioning of MSCs with TGF-β3 also induces a downregulation in these hypertrophic markers [42]. Parathyroid hormone and parathyroid hormone-related peptide (*PTHrP*) also delay chondrocyte hypertrophy through their regulatory actions on the *RUNX2* gene, which normally promotes hypertrophy [45,46]. In the present study, FGF-18 also upregulated *MEF2C*, a key osteogenic regulatory transcription factor, and osteogenic genes such as *ALPL*, *COL1A1 and BGLAP*. Osteocalcin (BGLAP) is a highly abundant bone protein secreted by osteoblasts, FGF-2 also stimulates production of BGLAP by chondrocytes [47]. The Gla domain of BGLAP interacts with calcium and hydroxyapatite and promotes bone mineralization and represents a useful early marker of bone formation. In contrast, FGF-2 promoted chondrogenesis through *ACAN*, *COL2A1*, *TGFB/TGFBR1* and *SOX9* in the present investigation, but did not induce osteogenesis.

### Upregulation of decorin & biglycan by FGF-2 & -18 in MSC micromass pellet cultures 7 their roles in matrix assembly, cell regulation, *in vitro* chondrogenesis, biomineralization & osteogenesis

In the present study *DEC* expression was strongly upregulated by FGF-2 and to a lesser extent by FGF-18 while *BGN* was strongly upregulated by FGF-18. Decorin and biglycan were immunolocalized in regions of the cell pellets where deposition of calcium and the novel CS sulphation motifs 7D4 and 4C3 were also evident. Decorin and biglycan have known roles in the regulation of collagen fibrillogenesis [48–50] and matrix stabilization by acting as linking and anchoring modules in collagenous matrices [51,52]. Decorin and biglycan have diverse functions in musculoskeletal tissues as modulators of tissue organization, cellular proliferation, matrix adhesion and response to growth factors and cytokines, and are key signaling molecules with an extensive repertoire of molecular interactions with growth factors and a number of receptors, which regulate cell growth and tissue morphogenesis [53–55]. Decorin regulates TGF-β1 bioavailability [56] and may have regulatory roles to play over TGF-β-dependant chondrogenesis [57]. Decorin and biglycan also have roles in biomineralization, which may explain their deposition in the central regions of the cell pellets in the present study [58,59]. Osteoblastic differentiation is accompanied by a decreased expression of decorin, but continuous expression of biglycan. TGF-β1 inhibits decorin expression in osteoblasts at varying stages of differentiation, but not biglycan [60]. Decorin and biglycan have differing roles to play in mineralization and are widely distributed in bone where they are present as CS substituted forms of these SLRPs, whereas in looser connective tissues the *D*-glucuronic acid of the GAG side chains is epimerized to *L*-iduronic acid; this confers differing interactive properties to the glycosaminoglycan (GAG) side chains on small leucine-rich proteoglycans (SLRPs) [52,58].

### Deposition of perlecan in pellet cultures

FGF-2 and -18 both upregulated perlecan expression in the present study. Immunolocalization studies on the cell pellets indicated that in basal cultures perlecan had a widespread pericellular distribution in the cells of the outer regions of the pellets and also in hypertrophic cells deeper in the pellet recapitulating the distribution patterns of perlecan in developing AC [61–63]. Perlecan is an early chondrogenic molecule [24] and has important cell regulatory [64] and matrix-stabilizing properties [28]. FGF-2 and -18 both signal cells through perlecan which acts as a low-affinity FGF receptor in cartilage [20,64]. In the FGF-2 and -18 pellets, the distribution of perlecan was predominantly around the cells of the outer regions of the pellets with significantly lower perlecan levels in the central regions where deposition of calcium, decorin and biglycan was evident.

### Genes operative in chondrogenesis & osteogenesis & their regulation by FGF-2 & -18

Figure 9 summarizes information on chondrogenic and osteogenic genes operative in chondrocyte differentiation. This figure also shows that FGF-2 was a prominent pericellular proteoglycan expressed by articular chondrocytes whereas FGF-18 was produced by the prehypertrophic columnar and hypertrophic chondrocytes adjacent to the calcified cartilage of the growth plates. The major findings with genes which were up- or downregulated by FGF-2 and -18 are also provided. The primary effect of FGF-2 was to promote chondrogenesis, while FGF-18 promoted early chondrogenesis, delayed chondrocyte hypertrophy and expression of proosteogenic genes. Calcium deposition was evident centrally in the FGF-18 pellets in regions, which also displayed the novel CS sulphation motifs 7D4 and 4C3. Decorin and biglycan were also immunolocalized in close association with these components but were not positively shown to be substituted with these CS motifs although evidence exists that decorin and biglycan can bear these motifs [65], which are prominently expressed in the chondrocostal growth plate cartilage as it transitions to bone [61,66].

### Immunolocalization of FGF-2 & -18 in developmental joint tissue identifies the cell types responsible for FGF production & their principle areas of action

Immunolocalization of FGF-2 in ovine newborn knee and hip demonstrated a strong localization of FGF-2 in the bone marrow and pericellular matrix of the articular chondrocytes. FGF-2 has known roles in the maintenance of stromal stem cells as a slowly recycling quiescent cell type and promotes stem cell viability. FGF-2 also has well-established roles in the regulation of chondrocyte metabolism and regulates cell proliferation and matrix production to ensure tissue homeostasis. While FGF-2 and -18 have similar properties in terms of the stimulation of chondrocyte proliferation and matrix production, FGF-18 has a more prominent expression pattern in the columnar prehypertrophic and terminally differentiated hypertrophic chondrocytes of the epiphyseal growth plate, which is consistent with its roles in chondrocyte maturational processes. Thus the immunolocalization patterns of FGF-2 and -18 in newborn and hip joint tissues observed in the present study were consistent with FGF-2 having a prominent role in AC chondrogenesis while FGF-18 had chondrogenic properties but also regulated growth plate chondrocytes, stimulating terminal chondrocyte differentiation and the expression of osteogenic regulatory genes in the transitional costochondral tissues at the bone-growth plate cartilage interface. These findings are consistent with our proposal that FGF-2 and -18 can be used to precondition bone marrow MSCs to select for chondrogenic and osteogenic cell lineages, which may have improved efficacy for the repair of cartilage and cartilage-bone defects. Studies already support the concept that FGF-2 primes adult bone marrow MSCs for enhanced chondrogenesis [22,67–68], however, the use of FGF-18 in similar studies is relatively unexplored. The present study has hopefully provided sufficient supportive evidence for the further exploration of FGF-2 and -18 as therapeutic agents to improve the application of adult MSCs in cartilage repair and in biointegrative applications on costochondral defects.

## Conclusion

The major conclusions from this study were that FGF-2 and -18 promoted chondrogenic differentiation of marrow-derived chondroprogenitor cells early in pellet culture with FGF-2 promoting matrix production and cellular proliferation, while FGF-18 delayed hypertrophy and also supported expression of osteogenic genes. Immunolocalization of FGF-18 in newborn ovine articular and growth plate cartilages clearly showed that FGF-18 was a pericellular matrix component of articular chondrocytes but was particularly strongly expressed by the hypertrophic growth plate chondrocytes of hip and knee joints. A recent study from our laboratory supported a prominent role for FGF-18 in the establishment and maturation of the vertebral ossification centers during fetal human spinal development [69]. FGF-2 and -18 also supported chondrogenesis but delayed hypertrophy, these therefore represent two useful growth factors which can be used to selectively target the marrow-derived stromal cells to develop chondrogenic and osteogenic cell lineages. Selective induction of such MSC cell lineages may well be of application in regenerative strategies maximizing matrix replenishment (FGF-2 lineages) and osteo-integration/osteogenesis (FGF-18 lineages) in tensional and weight-bearing connective tissues such as the spine.

## Future perspective

MSCs show tremendous potential in cell-based tissue regenerative strategies. Cartilaginous tissues, including the intervertebral disc suffer from a poor spontaneous repair capability. The application of MSCs in repair strategies in these tissues has shown promise in preclinical *in vitro* and experimental animal models of tissue repair. The development of MSC cell lineages directed to chondrogenesis and osteogenesis should improve the efficacy of these cells in specific regenerative strategies on cartilaginous tissue defects, which otherwise represent a considerable clinical problem.

**Table 1.** **Validated ovine primers used for reverse transcription PCR analyses.**

**Gene**	**Species accession #**	**Sequence (forward and reverse)**	**Melt temp (°C)**	**Product (bp)**
*COL1A1*	*Ovis aries* AF129287	F – ATC CCT GGA CAA CCT GGA CTT C R – TCA TCA TAG CCG TAA GAC AAC TGG	57	107
*COL2A1*	*Bos taurus* X02420	F – TGA CCT GAC GCC CAT TCA TC R – TTT CCT GTC TCT GCC TTG ACC C	55	154
*COL10A1*	*B. taurus* X53556	F – AAT GCC TGT GTC TGC TTT CAC TG R – GGA GTT GGG GAA TGC CTT TTC	56	454
*ACAN*	*B. taurus* U76615	F – TCA CCA TCC CCT GCT ACT TCA TC R – TCT CCT TGG AAA TGC GGC TC	58	105
*ADAMTS4*	*B. taurus* NM181667	F – AAC TCG AAG CAA TGC ACT GGT R – TGC CCG AAG CCA TTG TCT A	60	209
*ADAMTS5*	*B. taurus* AF192771	F – GCA TTG ACG CAT CCA AAC CC R – CGT GGT AGG TCC AGC AAA CAG T	55	97
*TGFB1*	*O. aries* X76916	F – CCC ACA GAG AGG AAA TAG AAG GC R – TGA AGC AGT AGT TGG TGT CCA GG	60	234
*TGFBR1*	*O. aries* AY656799	F – GGT CGT TTT GGA GAA GTT TGG C R – CCA TTG TCT TTG TTG TCC GCT G	55	173
*SOX9*	*O. aries* EE838714.1	F – CAT CAC CGC CTT GTC GTT AGA C R – AGA ATC TCC ATC GTC CTC CAC G	57	121
*HSPG2*	*B. taurus* XM582024	F – ATG GAT ACC ACC GTC ACC TAC G R – CTG TGT TGT GCT GGC AGT TCA G	60	229
*COMP*	*B. taurus* XM879092	F – CAA CTG TCC TCA GAA GAG CAA CG R – CCA CCT TGT CCG CAT CAA AG	60	317
*ALPL*	*B. taurus* NM176858	F – GCA GGC AGA GAG CAT AGA C R – TAT GGA CAG ACT TGG GG	53	287
*MEF2C*	*O. aries* NM001159277	F – TAT GCA AGC GAA ATC TCC TC R – AGT TGC TAC GGA AAC CAC TG	53	172
*BGLAP*	*O. aries* DQ418490	F – GCA CAG CCT TCG TGT CCA AG R – TAG AGC AGC GGG GAT GAT GG	61	294
*DCN*	*O. aries* AF125041	F – CCA AAG TGC GAA AGT CTG TGT TC R – CAG CAA TGC GGA TGT AGG AGA G	55	138
*BGN*	*O. aries* AF034842	F – TGA TTG AGA ACG GGA GCC TGA G R – TTT GGT GAT GTT GTT GGT GTG C	56	143
*MMP2*	*O. aries* AF267159	F – TGC TAC CAC CTC CAA CTA CGA TG R – GTG CCA GTA TCA ATG TCA GGG G	60	240
*MMP3*	*B. taurus* AF135232	F – TCC CCC AGT TTC CCC TAA TG R – GAT TTC TCC CCT CAG TGT GCT G	58	124
*MMP13*	*O. aries* AY091604	F – TGA CAG GCA GAC TTG ATG ATA AC R – CAT TTT GGA CCA CTT GAG AGT TC	58	113
*TIMP1*	*O. aries* S67450	F – GGT TCA GTG CCT TGA GAG ATG C R – GGG ATA GAT GAG CAG GGA AAC AC	57	265
*TIMP3*	*B. Taurus* NM174473	F – CTT CCT TTG CCC TTC TCT ACC C R – TCT GGT CAA CCC AAG CAT CG	57	286

Primers were authenticated by melt curve analysis and by sequencing.

F: Forward; R: Reverse.

**Executive summary**

**The utility of the development of chondrogenic and osteogenic mesenchymal stem cell lineages**

The availability of ‘off the shelf’ cell lineages of defined phenotype would be a very useful therapeutic tool for specific tissue repair applications which involve chondrogenesis or the integration of cartilage with bone. The chondrogenic and osteogenic mesenchymal stem cell lineages developed in the present study may be useful in such applications and deserve further evaluation.

**The requirement for improved therapeutic options in the treatment of IVD degeneration**

IVD derangement leading to the generation of LBP is a particularly debilitating musculoskeletal condition which deserves better therapeutic treatment methods. mesenchymal stem cells have shown tremendous promise for the treatment of IVD degeneration in laboratory based animal studies and in preclinical trials indicating that the cell lineages developed in this study should be evaluated further in appropriate large animal models and preclinical trials for the treatment of this condition.
